# A comparison of genomic copy number calls by Partek Genomics Suite, Genotyping Console and Birdsuite algorithms to quantitative PCR

**DOI:** 10.1186/1756-0381-4-8

**Published:** 2011-04-13

**Authors:** Britney L Grayson, Thomas M Aune

**Affiliations:** 1 Department of Microbiology and Immunology, Vanderbilt University School of Medicine, Nashville, TN 37232, USA; 2 Department of Medicine, Vanderbilt University School of Medicine, Nashville, TN 37232, USA

**Keywords:** Copy number variation, Calling algorithm, PCR

## Abstract

**Background:**

Copy number variants are >1 kb genomic amplifications or deletions that can be identified using array platforms. However, arrays produce substantial background noise that contributes to high false discovery rates of variants. We hypothesized that quantitative PCR could finitely determine copy number and assess the validity of calling algorithms.

**Results:**

Using data from 29 Affymetrix SNP 6.0 arrays, we determined copy numbers using three programs: Partek Genomics Suite, Affymetrix Genotyping Console 2.0 and Birdsuite. We compared array calls at 25 chromosomal regions to those determined by qPCR and found nearly identical calls in regions of copy number 2. Conversely, agreement differed in regions called variant by at least one method. The highest overall agreement in calls, 91%, was between Birdsuite and quantitative PCR. Partek Genomics Suite calls agreed with quantitative PCR 76% of the time while the agreement of Affymetrix Genotyping Console 2.0 with quantitative PCR was 79%.

**Conclusions:**

In 38 independent samples, 96% of Birdsuite calls agreed with quantitative PCR. Analysis of three copy number calling programs and quantitative PCR showed Birdsuite to have the greatest agreement with quantitative PCR.

## Background

Copy Number Variants (CNVs) are defined as amplifications or deletions of >1 kilobase segments of the genome [1,2]. Gene duplications were first identified in the pathogenesis of Charcot-Marie Tooth disease in the 1980s; a copy number (CN) amplification of the PMP22 gene was shown to be sufficient to cause disease [3]. These regions of variance were thought to be rare and when the human genome was published, variance amongst humans was primarily attributed to base-pair level single nucleotide polymorphisms (SNPs) [4,5]. However, CNVs were discovered to be present and widespread in the genome shortly thereafter [1,2]. These variants are generated during normal recombination events, leading to inherited CNVs, as well as somatically throughout life in rapidly dividing cells [6-8]. CNVs can directly influence gene expression through dosage effects where more copies of the gene produce greater expression, and also by altering transcriptional regulation in the genome, both in the region of variance itself and also in regions up to 1 megabase away [9-11].

CNVs can be detected by fluorescence in situ hybridization, bacterial artificial chromosome arrays, genome-wide SNP arrays or direct quantitative PCR (qPCR) in a genomic region of interest. One example of a genome-wide array is the Affymetrix SNP 6.0 array, with close to 1 million probes for determining SNPs across the genome and an additional ~1 million probes specifically designed to assess CN. Data from these arrays can be transformed into CN using any of a number of methods, including defined threshold intensity cut-offs and complex statistical algorithms such as circular binary segmentation and the Hidden Markov Model [12]. Circular binary segmentation determines copy number by looking for points along the genome where the copy number changes, inferring a loss or gain of copy number based on the change in intensity value [13]. Hidden Markov Models, like the one used in PennCNV, use the normalized average intensity along with the relative ratio of two potential alleles at each probe combined with the distance between probes and the population frequency of alleles to determine one of 6 possible states of copy number (loss of 1 copy, loss of 2 copies, normal state, normal state with loss of heterozygosity, single copy duplication or double copy duplication) [14].

These calling methods are built in to user accessible programs like Partek Genomics Suite, Affymetrix's Genotyping Console 2.0 and Copy Number Analysis Tool, and Birdsuite software developed by the Broad Institute at Harvard, among others [15]. A recent analysis evaluating the performance of seven CN calling algorithms - including circular binary segmentation[13], PennCNV[14], CNVFinder[16], cnvPartition, gain and loss of DNA[17], Nexus segmentation methods Rank and SNPRank and QuantiSNP[18]- found QuantiSNP outperformed other methods and had the highest statistical power to detect CNVs [19]. However, this comparative analysis was based on consensus of calls amongst the methods and did not assess a non-array reference, like qPCR, that might determine the accuracy of the calls.

Due to concerns about accuracy when only one calling method is used, CNVs have been associated with a number of diseases and states based on the use of multiple algorithms with a consensus call made for each genome region [20], or from just one algorithm paired with additional validation like qPCR or multiple ligation-dependent probe amplification methods [21-25]. In addition to the programs and methods already mentioned, new methods continue to be introduced in the literature [26,27].

We hypothesized that qPCR could finitely determine copy number and through this process, assess the validity of a calling algorithm. To test this hypothesis, we took data from 29 Affymetrix SNP 6.0 arrays and called copy numbers across the genome using three separate programs: Partek Genomics Suite, Affymetrix Genotyping Console 2.0 and Birdsuite. We compared the array calls at 25 individual chromosomal regions with copy number calling in the same genomic DNA samples by qPCR. The highest overall agreement in calls, 91%, was between Birdsuite and quantitative PCR. Partek Genomics Suite calls agreed with quantitative PCR 76% of the time while the agreement of Affymetrix Genotyping Console 2.0 with quantitative PCR was 79%.

## Methods

### Patient Recruitment

Patients were recruited by the Clinical Research Center at Vanderbilt University. These studies were approved by the Institutional Review Board of Vanderbilt University and all subjects provided written informed consent.

### Affymetrix SNP 6.0 Arrays

Peripheral blood was drawn into a Vacutainer Venous Blood Collection Tube (BD Catalog #367861) containing EDTA. Equal volume of lysis buffer (0.32 M Sucrose, 10 mM Tris-HCL, 5 mM MgCl2, 0.75% Triton X-100, pH 7.6) and 2× volumes of dH20 were added to each. Samples were centrifuged and resuspended in lysis buffer. After a second centrifugation, the pellet was resuspended in proteinase K buffer (20 mM Tris-HCl, 4 mM Na_2_-EDTA, 100 mM NaCl, pH 7.4) and proteinase K (20 mg/ml) was added to the solution. Samples were incubated for 1 h at 55°C, cooled on ice and 5.3 M NaCl was added. Samples were centrifuged, supernatants kept and added to cold isopropanol and incubated for 30 minutes. Finally, genomic DNA was centrifuged and the pellet was washed twice with 70% ethanol. Genomic DNA was dissolved in Tris-HCl (pH 8.0) and hybridized to the Affymetrix Genome-Wide Human SNP Array 6.0 (Santa Clara, CA) according to the manufacturer's protocol. Following scanning, arrays were checked for quality using Affymetrix Genotyping Console. Arrays with a Contrast QC less than 0.4 were removed from further analysis.

### Copy Number Analysis

Genotypes and CN were called using three different methods [Additional file 1]. The data were loaded into Partek Genomics Suite, quantile normalized and compared to the Hap Map 6.0 baseline. The Partek algorithm assumes that the pooled data from Hap Map 6.0 represent an average CN of 2. CN calls were made by comparing intensity values of each test sample to the pooled reference sample. CNV regions were called based upon the presence of different intensity values across at least 3 consecutive probe sets. Data were also loaded into the Affymetrix program Genotyping Console 2.0. This program used a similar, but not identical, algorithm to Partek in that the unknown samples were compared to a Hap Map reference file with the assumption of pooled intensity representing CN = 2. CN was determined with reference to the GenomeWideSNP_6.hapmap270 file and CNVs were similarly called based upon the presence of different intensity values across at least 3 consecutive probes. Finally, data were inputted into Birdsuite v.1.5.3 and, in contrast to the previous programs, variant regions were called without a pooled reference file. Birdsuite uses SNP data from known high frequency alleles in the population to stratify intensity ranges corresponding to different copy numbers. Applying these known data to unknown samples allows for inference to an unknown sample without the use of either a singular or pooled reference file. As a further quality control step for Birdsuite, arrays with an overall call rate less than 98% were discarded from further analysis.

### Quantitative PCR Experiments

To validate the copy number of variant regions from the Affymetrix chip, primer assays were ordered from Applied Biosystems, either custom designed or selected from their inventoried stock of assays, all designed specifically to detect genomic copy number (Additional file 2). Reactions were run with 20 ng genomic DNA per the standard Applied Biosystems protocol in a 7300 Real Time PCR System. All samples were run in triplicate with a multiplexed RNase P or Hemoglobin-beta reference assay and copy number was called using ΔΔCt values calculated in Applied Biosystem's CopyCaller v.1.0. In cases where a calibrator sample with CN of 2 was not known, plates were calibrated to an average CN = 2.

Twenty-five individual assays were employed; assay names, chromosomal locations and probe sequences were:

Hs05814268-Chr2:240,032,088-TGTACGACAAACATCTTCTGCCCTC;

Hs03085145-Chr2:242,648,367-CGGGTAAGGAGCCTGGTACGGGTCG;

Hs03488384-Chr3:53,010,599-GTAGATGGCAGCTCACATTTACTGT;

PIK3CA_ANY-Chr3:180,366,781-CACGGCTCACATGTTC;

Hs03609602-Chr6:326964-GGGAATTTCTGAAGGGAGTTTCATA;

BC040327-Chr7:11,288,419-CAGAGAGATGAAAATCT;

T1D_Chr7_ANY-Chr7:24,002,710-CTGCCCTCTCAGCCCC;

Hs04989338-Chr7:133,441,893-TTCTCATCAAGGTATGTGGCTCATT;

Hs04351655-Chr8:51,195,001-TACTCAGGATATGCATTACATACTT;

Hs03694840-Chr8:144,776,429-CAGAGTCTACCAGAGAGGGTGTCCT;

T1D_Chr9_ANY-Chr9:16,930,899-CAGCCGCTATTTGCT;

Hs06372538-Chr13:53,784,055-TCAAGTAAGTGCTACAGCCAATAAT;

Hs03298240-Chr13:56,713,547-AAAGAAGATTTGAACAGAGCAAAGA;

Hs03172318-Chr15:32,555,299-AAATTTTCATTCGCAATATGAATCC;

Hs05443340-Chr16:3,104,308-TATCCAGGACTTTCCTGAGCTGGCA;

Hs05412190-Chr16:4,280,824-ATGCTGGCTGGACTGTTTCTGCTTT; C16v1.3_CCE924B_F:Chr16:18,557,305-CATGACCGTCTTCCAGAATGT; SIGLEC5_5_ANY-Chr19:56,824,268-CAGGACAGCCTTCCCC;

SIGLEC14_3_ANY-Chr19:56,838,253-CCCCACCACACCTGC;

SIRPB1_Mi7_ANY-Chr20:1,522,539-ATTTTTGGAGGCATGAAACT;

Hs04045482-Chr20:28,068,523-CATGGATTTAAGAGCAGAGTCATGG;

Hs04497042-Chr20:29,271,114-GAATACACTCGAATGCAATGGAATA; DDTL_3UTR_ANY-Chr22:22,643,636-TCCGTGCCCAATCATA;

Hs00010004-Chr22:22,713,888-GGCCGAATAAAGGGGTGGGGATCAT;

FAM19A5_ANY-Chr22:47,400,722-CCATGCGTGCAGTTTT.

Assays denoted by "Hs########" represent inventoried assays designed by Applied Biosystems. All others were custom-designed, also from Applied Biosystems. Custom designed primers were compared to the dbSNP database using the online NCBI tool (http://www.ncbi.nlm.nih.gov/SNP/snp_blastByOrg.cgi) to ensure there were no overlapping SNPs in these genomic regions.

## Results

Genomic DNA samples from 77 individuals were hybridized on Affymetrix SNP 6.0 Arrays. 29 of these samples were analyzed for CN using the Partek Genomics Suite, Genotyping Console 2.0 (GTC) and Birdsuite [see Additional file 1]. Array-based copy number calls). Of note, both Partek and GTC CN calls were determined using a pooled Hap Map comparison file. qPCR analysis was performed to determine CN at a total of 25 individual genomic regions across 12 chromosomes. The results were compared to the 3 sets of genome-wide calls made from the arrays (see Additional file 2, comparison of copy number calls).

### Invariant Regions

A number of regions were identified by one or more of the algorithms to have CN of 2 in all samples tested. We probed 16 of these "invariant" regions by qPCR and compared the results of the calls in each sample by each method (Table 1). There was vast agreement in CN calls in these regions. Of note, qPCR called 4 samples variant at chromosome 2 that were called CN of 2 by all three algorithms. Additionally, in a region on chromosome 8, Partek and GTC called all 8 regions a CN of 2 while Birdsuite called 2 samples variant. Those same 2 samples were also found to be variant by qPCR. Additionally, one sample was called variant by GTC on chromosome 9 but invariant, or CN of 2, by all other methods. All together, seven sample-region pairings were called variant by just one or two methods and invariant by the others while 209 sample-region pairings were uniformly called CN of 2 by Partek, GTC, Birdsuite and qPCR, representing nearly 97% agreement amongst all methods of CN calling in these 16 regions.

**Table 1:** Copy Number Calls at Invariant Regions of the Genome

Region	Partek	**GTC**^**a**^	Birdsuite	qPCR
2:240,032,088	28/0^b^	28/0	28/0	24/4

3:180,366,781	27/0	27/0	27/0	27/0

6:326,964	5/0	5/0	5/0	5/0

7:11,288,419	17/0	17/0	17/0	17/0

7:24,002,710	24/0	24/0	24/0	24/0

8:51,195,001	8/0	8/0	6/2	6/2

9:16,930,899	25/0	24/1	25/0	25/0

13:53,784,055	9/0	9/0	9/0	9/0

13:56,713,547	9/0	9/0	9/0	9/0

15:32,555,299	8/0	8/0	8/0	8/0

16:3,104,308	9/0	9/0	9/0	9/0

16:4,280,824	8/0	8/0	8/0	8/0

16:18,557,305	9/0	9/0	9/0	9/0

20:28,068,523	6/0	6/0	6/0	6/0

20:29,271,114	8/0	8/0	8/0	8/0

22:47,400,722	16/0	16/0	16/0	16/0

^a^GTC = Genotyping Console 2.0

^b^Values are represented as "CN = 2"/"CN = non 2"

### Variant Regions

Additional regions were identified as variant, containing numerous CNVs amongst the 29 samples. Nine of these regions were investigated by qPCR, 184 sample-region pairs in total, and the results produced by the three CN calling algorithms were compared by CN class, 0, 1, 2, 3 and 4 (Table 2). Region 1 on chromosome 2 produced an identical group of CN calls amongst each of the 4 methods. GTC, Birdsuite and qPCR also produced identical CN calls in Region 2. Additional regions 3-9 did not show such similarity in CN calls between the 4 methods but these comparisons suggested that agreement was highest when Partek calls were compared to GTC calls or when Birdsuite calls were compared to qPCR. Regions 3, 4 and 8 produced a very similar breakdown of calls in Partek and GTC while region 7 was nearly identical, with 7 samples being called amplifications by both programs. Regions 3, 4, 6, 7 and 9 showed similar calls by both Birdsuite and qPCR. These analyses indicate that some patterns of agreement were observed amongst the different methods of CN calling.

**Table 2:** Comparison of copy number calls at variant regions

CN	0	1	2	3	4	0	1	2	3	4	0	1	2	3	4
	Region 1					Region 2					Region 3				

	Chr2:242,648,367					Chr3:53,010,599					Chr7:133,441,893				

Partek	0^b^	4	24	0	0	0	1	24	0	0	0	1	5	0	0

GTC	0	4	24	0	0	1	3	21	0	0	0	1	4	1	0

Birdsuite	0	4	24	0	0	1	3	21	0	0	1	3	2	0	0

qPCR	0	4	24	0	0	1	3	21	0	0	1	3	2	0	0

	Region 4					Region 5					Region 6				

	Chr8:144,776,429					Chr19:56,824,268					Chr19:56,838,253				

Partek	0	0	8	0	0	0	0	13	13	0	0	0	7	6	0

GTC	1	0	7	0	0	0	0	17	3	6	0	0	6	3	4

Birdsuite	1	2	5	0	0	0	0	26	0	0	0	2	11	0	0

qPCR	1	2	5	0	0	1	6	19	0	0	0	2	11	0	0

	Region 7					Region 8					Region 9				

	Chr20:1,522,539					Chr22:22,643,636					Chr22:22,713,888				

Partek	0	4	13	7	0	0	0	23	5	0	0	0	14	12	0

GTC	0	4	13	0	7	1	0	24	3	0	1	1	16	6	2

Birdsuite	17	7	0	0	0	12	11	5	0	0	2	13	11	0	0

qPCR	18	6	0	0	0	0	0	28	0	0	2	14	10	0	0

^a^GTC = Genotyping Console 2.0

^b^Values are numbers of samples called each copy number at each region.

### Agreement of CN calls

**Figure 1:**
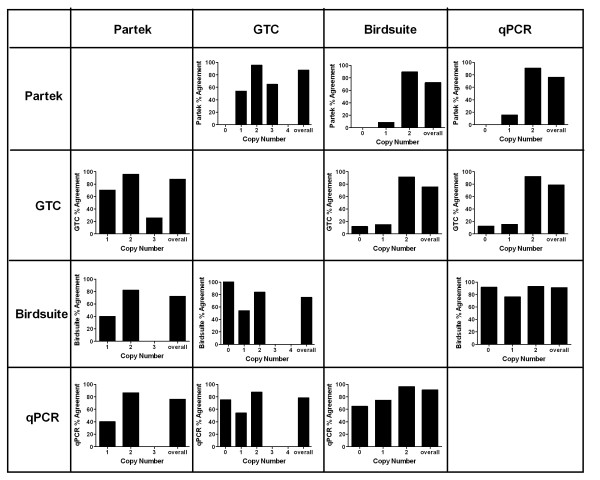
**Agreement of CN calls made by Partek, GTC, Birdsuite and qPCR**. CN calls were analyzed on a sample-by-sample basis across 25 individual chromosomal regions. Results were sorted according to the CN called by the method named at the top of each column. Descending in each column are method-by-method comparisons. A total of 400 CN calls were considered in this analysis. Results are expressed as % agreement between any two methods of CN call determinations.

To determine exact agreement among the CN calling methods, 400 CN calls were compared on a sample-by-sample basis to determine agreement of each CN state (Figure 1). As previous analyses indicated, the highest agreement in every comparison was seen at CN = 2 among the individual CN states (0, 1, 2, 3, and 4). These agreements ranged from 82% to 96% and greatly influenced the overall agreements of each comparison. Discordance among calls from each method was found by comparing the variant calls (CN of 0, 1, 3 or 4).

When Partek called a CN of 1, GTC also called the sample a CN = 1 70% of the time. However, when GTC called a sample region CN = 1, Partek correctly called that region a CN = 1 54% of the time. There was no agreement between GTC and Partek at CN of 0 because Partek did not call any CN = 0 in any of the tested regions. Partek showed less than 50% agreement with variant calls in both Birdsuite and qPCR. The overall agreement of GTC with Partek was 88%, between Birdsuite and Partek was 72% and qPCR CN calls agreed with Partek CN calls 76% of the time.

When GTC called a CN of 0, Birdsuite also called a 0 100% of the time while the agreement with qPCR was 75%. At copy number of 1, Birdsuite and qPCR had an identical call in less than 60% of samples. Conversely, when Birdsuite or qPCR made a copy number call of 0 or 1, GTC reported the same call in those samples less than 20% of the time. Overall agreement of Birdsuite with GTC was 76% and qPCR and GTC agreed in 79% of the samples.

When Birdsuite called a CN of 0 or 1, the agreement with Partek was 0% and 9%, respectively. Birdsuite variant calls agreed with GTC's calls at slightly higher rates, 12% for CN = 0 and 15% for CN = 1. The agreement between Birdsuite and qPCR, however, was 65% for CN of 0 and 75% for CN of 1. Of note, the majority of the disparate calls in this comparison came from region 8 (Table 2), where Birdsuite called 23 samples to be CN deletions while qPCR determined them to be CN = 2. While GTC and qPCR showed high agreement at CN of 0, the agreement at CN = 1 was 54%. The Birdsuite agreement with qPCR in copy number variant sample regions were thus the highest seen among any comparison of array-based calls with qPCR. The overall agreement of qPCR with Birdsuite was also the highest, at 91%.

Finally, when CN calling from array-based algorithms were compared to qPCR, GTC and Partek both showed variant agreements less than 20% of the time, while Birdsuite agreed with 92% of qPCR calls at CN = 0 and 76% of qPCR calls at CN = 1. Similar to the inverse comparison of qPCR calls to Birdsuite, when Birdsuite variant calls were compared to qPCR, region 5 (Table 2) showed 6 samples that were determined to be CN deletions by qPCR and called CN of 2 by Birdsuite, accounting for a large portion of the 24% error in calls at CN = 1. Overall, the highest agreement was found between qPCR and Birdsuite.

### Agreement of CN calls in a second, independent group

**Figure 2:**
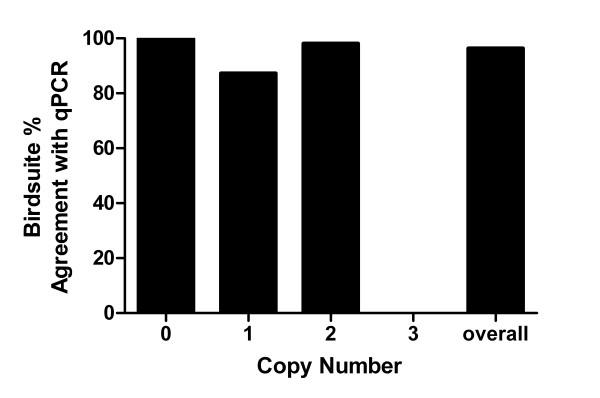
**Agreement between CN calls made by Birdsuite and qPCR**. Birdsuite CN calls were compared to qPCR copy number calls at 18 distinct chromosomal regions on 10 chromosomes. A total of 387 data points were considered in the analysis. At CN = 0, 12/12 samples agreed, at CN = 1, 48/55 samples agreed, at CN = 2, 313/319 samples agreed and at CN = 3, 0/1 sample agreed. Results are expressed as % agreement between any the two methods of CN call determinations.

We next assessed if the high percent agreement between Birdsuite and qPCR was reproducible in a second independent group of samples. Data from 38 additional Affymetrix SNP 6.0 arrays were analyzed by Birdsuite to determine copy number calls across the genome (see Additional file 1: Array-based copy number calls). qPCR reactions were performed using 18 different assays investigating regions on 10 chromosomes to determine CN at each region. A total of 387 comparisons were made in this step (Table 3 and Figure 2). A total of 14 Birdsuite calls in 7 genomic regions did not agree with the CN call made by qPCR (Table 3). Six of these disparate calls were CN = 2, 7 of CN = 1 and 1 of CN = 3. Overall agreement at each CN was also determined (Figure 2). Birdsuite and qPCR agreed on 100% of CN = 0, 87% of CN = 1 and 98% of CN = 2. Of note, there was 0% agreement in 1 sample called a CN = 3 by qPCR. The overall agreement of Birdsuite with qPCR was 96%, better than the overall agreement rate from the first analysis (91%).

**Table 3:** Birdsuite Agreement with qPCR calls in 18 genomic regions

Region	0	1	2	3
2:242,648,367	0 (0)	1 (0)	36 (0)	0 (0)

3:53,010,599	1 (0)	2 (1)	30 (0)	0 (0)

3:180,366,781	0 (0)	0 (0)	16 (0)	0 (0)

7:11,288,419	0 (0)	0 (0)	26 (0)	0 (0)

7:24,002,710	0 (0)	0 (0)	16 (0)	0 (0)

7:133,441,893	2 (0)	14 (0)	6 (0)	0 (0)

8:51,195,001	1 (0)	11 (2)	13 (0)	0 (0)

8:144,776,429	1 (0)	5 (2)	10 (2)	0 (0)

9:16,930,899	0 (0)	0 (0)	16 (0)	0 (0)

13:53,784,055	0 (0)	0 (0)	18 (0)	0 (0)

13:56,713,547	0 (0)	0 (0)	13 (4)	0 (0)

13:71,376,533	7 (0)	12 (1)	1 (0)	0 (0)

15:32,555,299	0 (0)	3 (0)	16 (0)	0 (0)

16:3,104,307	0 (0)	0 (0)	18 (0)	0 (0)

16:4,280,826	0 (0)	0 (1)	15 (0)	0 (0)

16:18,557,305	0 (0)	0 (0)	26 (0)	0 (0)

20:29,271,114	0 (0)	0 (0)	8 (0)	0 (1)

22:47,400,722	0 (0)	0 (0)	29 (0)	0 (0)

^a^Values are represented as the number of calls that "agree(disagree)"

## Discussion

A total of 77 peripheral blood genomic DNA samples were analyzed for CN on Affymetrix SNP 6.0 Arrays. CN calls for 29 of these samples were determined by three different methods: Partek Genomics Suite, GTC and Birdsuite. Calls at 25 genomic regions were also determined by qPCR in these same samples. Comparison of these CN calls shows that all 4 methods agreed when the CN call is 2. However, there is considerably less agreement when the CN calls identify variant regions or CNVs. One way to determine one singular CN call for each region would be pooling the array CN calls from each algorithm to arrive at a consensus call. However, the disagreement amongst variant calls by Partek, GTC and Birdsuite seen in this sampling prohibit arriving at a clear consensus.

Table 2 shows how using different combinations of algorithms can result in widely variant CN calls in the same samples. If the combination of Partek and GTC is used, whole genome scans would determine Region 5 to have increased CN and Region 7 to be split between copy number duplications and loss to CN = 1. Upon investigation of these specific regions in additional samples by qPCR, the data are not consistent. Data from qPCR analysis demonstrate that Region 5 is actually a CN loss while Region 7 has a CN = 0 in many of the samples called a CN gain by the algorithms. One reason for the overlapping calls resulting from two independent algorithms is that both Partek and GTC use a pooled Hap Map reference file. This suggests that if multiple "different" algorithms are used in an attempt to create more accurate data but each algorithm uses a version of the pooled Hap Map reference, the resulting CN calls may appear to be in agreement but this agreement is based on the similarity of the reference file and not the actual CN call of a region in the test sample as it might be determined by qPCR.

To take the consensus of the 3 algorithms analyzed in this paper to mine array data for potential variants of interest would similarly result in data that could not be verified by qPCR. In this case, the agreement between 2 of the 3 algorithms using the same reference file could potentially exclude a different call made by the algorithm without a reference file. While qPCR does not represent a feasible method for screening genomes for CNV for both technical and financial reasons, any CNV region of interest discovered using array data or pooling of CN calls ultimately needs to be validated by a non-array method, like qPCR. When each of the 3 methods are compared to qPCR, the highest agreement both in variant calls and overall calls is between qPCR and Birdsuite.

CN calls made by each algorithm- Partek, GTC and Birdsuite, on each array are subject to a number of assumptions. CN calls are calculated in GTC and Partek by using a reference file or baseline. This reference file is generally composed of pooled CN data such that the average intensity of the group is assumed to be CN of 2. However, in the 77 arrays analyzed we discovered numerous regions to be variant in greater than 80% of samples. Pooling these arrays and assuming a CN of 2 would therefore skew results. In contrast, Birdsuite uses a unique method to determine copy number. The Broad Institute has previously characterized copy number polymorphisms by determining those CNVs present in greater than 5% of the Hap Map population [28]. Birdsuite uses known intensity value-copy number references at the 1,320 copy number polymorphisms to infer CN in the remaining portions of the genome [15]. However, no algorithm can completely escape the problem of background intensity on the array and the risk for type I and type II errors that come with the sampling of intensity values at nearly 2 million probes.

CN determined by qPCR is not without assumptions. Calls are made using the ΔΔCt calculation with the first comparison coming between the test assay Ct value and a multiplexed reference assay Ct value. Reference assays exist for genes known to be CN invariant, or to always have exactly 2 copies of the gene in the genome. The second comparison is made between the test assay-reference assay value and that same value for a calibrator sample, known to have a CN = 2 in the test region. If the calibrator sample is not a CN of 2, the data would be skewed in the direction of the actual CN of the calibrator sample. qPCR, however, does not have the problem of additional background noise and is also immune to multiple sampling errors. For these reasons, qPCR is considered to be the standard in determining copy number.

## Conclusions

The algorithm employed by Birdsuite to call CNs across the genome closely agrees with the qPCR determinations of copy number. When all 787 comparisons from these data are considered, the overall agreement is 94%. For this reason, the use of the Birdsuite algorithms, in combination with PCR validation, generated the most reproducible CN calls in this group of patient samples. Of note, more recent versions of Genotyping Console now employ the Birdsuite algorithms to determine CN.

## List of abbreviations used

CN: copy number; CNV: copy number variation; GTC: Genotyping Console 2.0; qPCR: quantitative polymerase chain reaction; SNP: single nucleotide polymorphism

## Competing interests

The authors declare that they have no competing interests.

## Authors' contributions

BG helped to conceive the study, carried out the qPCR experiments, called CN by GTC, compared all data, interpreted the results and drafted the manuscript. TA helped to conceive the study, participated in the design of the study and edited the manuscript. Both authors read and approved the final manuscript.

## Supplementary Material

Additional file 1 **Array-based copy number calls**. Raw CN calls for 29 samples in Partek, 29 samples in GTC and 77 samples in Birdsuite. Calls are organized by sample across the genome from chromosome 1-24. Click here for file

Additional file 2 **Comparison of copy number calls**. CN comparisons between Partek, GTC, Birdsuite and qPCR used to determine agreement in the tables and figures presented herein. Click here for file
